# Nutritional, Physicochemical, and Sensorial Evaluation of Flat Bread Supplemented with Quinoa Flour

**DOI:** 10.1155/2019/4686727

**Published:** 2019-03-03

**Authors:** S. A. El-Sohaimy, M. G. Shehata, Taha Mehany, M. A. Zeitoun

**Affiliations:** ^1^Food Technology Department, Arid Lands Cultivation Research Institute, City of Scientific Research and Technological Applications, Egypt; ^2^Food Science and Technology Department, Faculty of Agriculture (Saba Basha), Alexandria University, Egypt

## Abstract

The purpose of the present research was to develop novel flat bread supplemented with quinoa flour to raise its nutritional quality and functional properties. Furthermore, evaluation of the quality of developed bread was realized with blends at 5, 10, 15, 20, 25, and 30% of quinoa flour. Chemical composition of supplemented flat bread was determined. Several properties on dough (water absorption, dough development time, stability time, elasticity, and extensibility) and their corresponding characteristics (loaf specific volume, baking loss, roundness, height, baking time, hardness, cohesiveness, springiness, resilience, gumminess, and chewiness) were then evaluated. The protein content in bread-based quinoa blends was significantly increased gradually with increasing the percentage of quinoa flour from 12.12±0.63% in control to 15.85±0.065% in 30% quinoa flour. Also, the amino acids content was increased with increasing the percentage of quinoa flour. Mineral contents in 30% quinoa flour blend such as sodium, potassium, magnesium, calcium, iron, copper, manganese, and zinc were higher than other ratios and control bread (100% wheat flour). Rheological properties of supplemented bread such as specific volume, appearance, crust and crumb texture, aroma-odor, and colour were evaluated and found to be excellent. Physicosensory characteristics of the bread fortified with quinoa flour were evaluated and the most of panelists accepted and preferred the bread supplemented with quinoa flour more than control. The obtained unique nutritional, physicochemical, and organoleptic characteristics of quinoa flour-based flat bread open a new promising prospect for utilization of quinoa flour in an industrial scale for treatment and/or prevention of malnutrition in developing counties.

## 1. Introduction

Functional foods are defined as any component or substance of a food that display health benefits, including the prevention and treatment of diseases [1]. The main functional components of foods are fibers, proteins, polyunsaturated fatty acids, phenolic compounds, prebiotics, and probiotics [2, 3]. In current years, the chemical composition, nutraceutical applications, and processing effects of quinoa have been investigated and described [4–7]. According to the recommendations announced by the FAO/WHO/UNU, quinoa (*Chenopodium quinoa *Wild, family Amaranthaceae) has well-balanced protein and amino acid content that could develop dietary protein balance when utilized by itself or mixed with cereal grains [8]. Quinoa is an Andean pseudo cereal. Once known to the Incas as “mother of all grains”, today quinoa is receiving increasing attention because of its high nutritional quality, mainly the protein content, and also as a valuable source of micronutrients [9]. Quinoa seeds are higher in protein than usual wheat and corn seeds, ranging from 12 to 18%. Unlike wheat that is low in lysine, quinoa includes a well-balanced set of necessary amino acids, making it an unusually complete plant protein source for humans. Furthermore, quinoa is a great source of phosphorus and dietary fiber. It is high in magnesium and iron contents as well as in vitamins such as vitamin E and those of the group B [10, 11]. Quinoa is gluten-free and deemed simple to digest. Celiac and lactose-intolerant subjects should be also quinoa consumers because of its gluten-free nature and its wealthy protein levels, similar to that of milk casein quality [12]. However, the flat bread making ability of quinoa seeds and wheat flour blends are not common in the literature. Because of its low baking character, which is due to the lack of gluten, quinoa flour can only partly replace wheat flour in bread production and other baked products. Turkut et al. [13] revealed that quinoa flour might be considered a good alternative for gluten-free pan bread making. The sensory evaluation of the appearance, texture, and flavour displays the products to be good acceptable [14]. However, supplementation of wheat flour dough with quinoa flour [15] appeared in nutritionally improved bread with sensory acceptance. Clearly, according to the many publications, the bread making capacity of quinoa seeds and wheat flour blends has not been reported yet. The objective of the present study was to develop high-quality flat bread based on quinoa flour. Furthermore, physiochemical and rheological properties and sensory evaluation of produced flat bread fortified with quinoa flour.

## 2. Material and Methods

Quinoa seeds (*Chenopodium quinoa* Wild, var. NAT OIL1) were obtained from the Egyptian Company for Natural Oils, Cairo, Egypt. The plant was cultivated in Egypt for over seven years till now on Cairo-Ismailia desert road. The collected seeds were cleaned of foreign materials and stored at room temperature (25 ± 2°C) for further analysis.

### 2.1. Flour Preparation

Quinoa flour was prepared according to Vilche et al. [16] with some modifications to remove saponin. Whole seeds were washed twice with cold water; then seeds were soaked in the alkaline solution for 10–20 min and then rinsed with 1% citric acid solution for 10 min. The cleaned seeds were washed with water until there was no foam as an indication of saponin removal from the seeds hull. Later saponin-free seeds were overnight oven-dried at 45±1°C. During drying treatment, the seeds were spread in a thin layer to avoid germination process and any further contamination. Finally, treated whole seeds were ground into flour using Miller (KARIZMA- JX-1000A) and kept at 5°C for further analyses.

### 2.2. Flat Bread Preparation and Recipe

Flat bread formulations using quinoa flour in different ratios are given in Table 1. Flat bread making was prepared according to Yaseen et al. [17]. Wheat flour blends with of 0, 5, 10, 15, 20, 25, and 30% of quinoa flour were used for making flat bread. The dry material individually blended to be homogenized (1.5 gm yeast and 1 gm salt) per 100 gm of flour blend. Water was added to blends according to the water absorption of farinograph. The blends were manually mixed for 15 minutes to form dough. The dough was kept at 25± 2°C, 85% RH, for 30 min. Dough pieces (100 g) were then flatted by using woody roll to round- flat bread (flat bread shape) about 20 cm diameter. Proofed at 25± 2°C, 85% RH, for 25 min to complete fermentation. Then loaves baked at 450°C in mechanical gas oven for 1-2 min. The flat bread loaves were allowed to cool and then packed in polyethylene bags.

### 2.3. Determination of Farinograph Characteristics of Blended Dough

Mixing properties of dough were evaluated using farinograph (Brabender OHG, Duisburg, Germany) according to AACC methods No. 54-10 [18]. In the farinograph, dough was prepared under standardized conditions from 300 g of wheat flour. Farinograph data including water absorption (percentage of water required to yield dough consistency of 500 BU (Brabender Units)), arrival time, dough development time (time to reach maximum consistency), and stability time (time during dough consistency is at 500 BU and degree of softening), centered on a 500-BU line. Dough types from wheat flour with the addition of variable quantities of quinoa flour (5, 10, 15, 20, 25, and 30%) were prepared from 300 g of these blends.

### 2.4. Determination of Extensograph Characteristics of Blended Dough

The standard extensograph method has been traditionally used to evaluate rheological properties of dough by providing information on dough strength (resistance to extension) and extensibility. Brabender extensograph (Brabender OHG, Duisburg, Germany) gave the resistance to constant deformation after 50 mm stretching (R50), extensibility (E), ratio number (R50/E), and area under the beak for each blend were determined according to AACC [18] methods No. 54-21.

### 2.5. Physiochemical and Rheological Properties of Quinoa Flour-Based Flat Bread

#### 2.5.1. Baking Quality

The bread quality attributes for flat bread loaves were evaluated after cooling at room temperature. For each loaf, triplicates from different sets of baking were analyzed and averaged. The loaves were* weighed* using balance (Sartorius, Germany);* height and diameter* (roundness) for each loaf were also determined from each blend.* Bake loss* (moisture loss) of bread loaves after baking was determined according to Alvarez-Jubete et al. [19] by the following formula:

(1) $$\begin{array}{c} Baking\;\;loss\;\;\text{\%}=\left.\;\frac{\left(Wbb–Wab\right)}{Wbb}\;\right)X\;\;100 \end{array}$$

where Wbb is the weight of the loaf before baking and Wab is the weight of the loaf after baking and cooling.* Loaf volume* was determined by millet-seed displacement method [20] by taking a known mass of sample (weight of loaf) in a container and covered with seeds to totally fill the container and the increase in volume was noted and calculated as loaf volume.

#### 2.5.2. Moisture Content

The moisture content of loaves for each blend was measured in an oven after intervals of 0, 24, 48, and 72 hr. During storage in polyethylene bags at room temperature (25°C ±3) according to A.O.A.C. [21]. % Water retention after 3 days was calculated from the following formula:

(2) $$\begin{array}{c} \text{\%}\;\;Water\;\;retention=\frac{\text{\%}\;\;Moisture\;\;at\;\;3\;\;day}{\text{\%}\;\;moisture\;\;at\;\;0\;\;day}x\;\;100 \end{array}$$

#### 2.5.3. Proximate Analysis

The analysis of ash, crude fibre, total protein, and total lipids was carried out as described in AOAC [21]. The total nitrogen free carbohydrate (NFE) was calculated by the difference: {100- (proteins + lipids + moisture + ash)}.

#### 2.5.4. Determination of Minerals Content

For mineral analyses, quinoa seeds flour (0.5 g) was weighed and ashing in muffle at 550°C for 2 hours. Then the ashes were dissolved with 100 ml 1 M HCl and then filtrated by Whatman (No 1). Dissolved ash was analysed for iron, magnesium potassium, sodium, zinc, manganese, copper, and calcium contents by using methods of AOAC [21]. Inductively Coupled Plasma-Mass Spectrometer (ICP/MS), NEXION 300X series, was used to determine of minerals (mg/100gm). Argon gas was used for excitation of the element atom.

#### 2.5.5. Determination of Amino Acid Composition

Amino acid analysis was carried out using performance amino acid analyzer (AAA 400, INGOS Ltd. Czech Republic) according to Block et al. [22] and Spackman et al. [23]. Bread sample was weighed (100 mg) into a glass ampoule, 10 ml of 6 N (HCl) was added to the ampoule, and the contents were hydrolyzed in an oven at 110°C for 24 h. Oxygen was expelled in the ampoule by passing nitrogen gas through it. The excess of HCl was then removed from 1 ml hydrolyzed under vacuum at 80°C with the occasionally addition of distilled water and then evaporated to dryness. HCl free residue was dissolved in exact 2 ml of loading buffer (6.2 M, pH 2.2). The analysis was carried out with a gas flow rate of 0.5 ml/min at 60°C, and the reproducibility was 3%. The amino acid composition was calculated from the areas of standards obtained from the integrator and expressed as percentages of the total protein according to the following equation:

(3) $$\begin{array}{c} \text{\%}AA=\frac{\text{\%}Area\;\;under\;\;the\;\;peak\;\;X\;\;\text{\%}\;protein}{100} \end{array}$$

#### 2.5.6. Texture Analyses

Loaf texture was determined by using texture analyzer (Model CT310K Texture Analyzer, USA). Texture profile analysis was performed on central bread slices by TPA test with a 3mm cylindrical stainless steel (TA44 probe. Downward speed was 0.5 mm/s and upward speed 0.5 mm/s with a trigger load of 4.0 g. Texture TA-RT-KI/ Load cell: 10 kg). The TPA parameters were calculated according to Guinee, [24, 25] such as hardness (g) (force required for a pre-determined deformation), springiness (mm) originally called “elasticity” (rate at which a deformed sample returns to its original size and shape), and cohesiveness (originally the ratio of energies expanded in the first and second cycles or strength of internal bonds in the sample), resilience (a measure of how well a product fights to regain its original position, is a parameter similar to elasticity. But it is expressed as a ratio of energies instead of a ratio of distance), chewiness (energy needed to chew food until it is ready for swallowing), and gumminess (energy needed to disintegrate food until it is ready to swallow).

#### 2.5.7. Colour Analysis

Three loaves of bread from each blend (0, 5, 10, 15, 20, 25, and 30% quinoa flour) were analysed for crumb and crust colour using the Hunter machine (UltraScanvis: us vis 1310, Hunter Laboratory, USA). The Ultra Scan Vis Colour (illuminant/observers D65/10) was standardized (calibration) before reading the samples. And the (L, a and b) values were recorded where L is lightness, a is red/green, and b is yellow/blue. For the L value, the value closer to 100 indicates the whiter (lighter) and the value closer to 0 indicates black (darkness). The scale for (a) is -100 to 100+ (where -100 is green and 100+ is red). The scale for (b) is the same as (a), except -100 being blue and 100+ being yellow.

### 2.6. Sensory Evaluation

Evaluation of the baked loaves quality characteristics was carried out following cooling to room temperature. Sensory evaluation was performed by 10 panelists who were staff members of Food Technology Department, City of Scientific Research, Alexandria, Egypt. Loaves were randomly assigned to each panelist. The panelists were asked to evaluate each loaf for loaf shape, mouth feel, flavour, crumb texture, crumb colour, crust colour, and crust texture, through nine-point scale according to Ihekoronye and Ngoddy [25] with 9-1: dislike extremely (1); dislike very much (2); dislike moderately (3); dislike slightly (4); neither like nor dislike (5); like slightly (6); like moderately (7); like very much; (8) like extremely (9).

### 2.7. Statistical Analyses

The data was analysed using SPSS version 16, USA. One-way analysis of variance with p ≤ 0.05 was performed to identify significant differences among all studies parameters by Duncan's test. All experiments carried out in triplicate.

## 3. Results and Discussion

### 3.1. Characteristics of Dough

#### 3.1.1. Farinograph Properties

*Farinograph* is often used to evaluate the rheological properties of dough such as water absorption of flour and other characteristics of dough during mixing and establishing the flour behaviour during the bread making process.  Table 2 showed the rheological properties of different blends of wheat and quinoa flour. Water absorption was varied from 59.50±0.50% to 63.83±0.29% of control dough and 30% quinoa dough, respectively.* Water absorption* significantly increased with the increasing of quinoa flour. Differences in water absorption between control and blends of quinoa flour might be due to the different protein and fibre contents. High content of protein and fibre in quinoa flour resulted in high water absorption rate of dough. On the other hand, there were no significant differences in arrival time between control dough and blends made from different ratio of quinoa flour. These results are in agreement with the results of [26, 27]; they found that water absorption is gradually increased with the increasing of quinoa flour in wheat flour blends dough.

*Dough development time* of wheat flour supplemented with different blends of quinoa flour was significantly increased with the increasing the quinoa flour percent. The blends of 10 and 15% quinoa flour had the highest dough development time (4.83 min.) compared to all other blends while the wheat dough (control) showed the lowest dough development time (3.33±0.29 min). Dough development time of 30% quinoa flour was 4.16 ±0.30 min. Differences in dough development time of control and blends of quinoa flour might be due to differences in the physicochemical properties between the constituents of quinoa and those of the wheat flour, also the higher rate of water absorption by quinoa flours due to higher amount of soluble protein in quinoa flour might result in longer dough development time. The results of the present work are in agreement with previous results [27], which declare that the addition of quinoa flour to wheat flour increases the dough development time. The starch constituents of quinoa flour might play the most important role in the prolong the time of the dough development.

*Stability value* is an index of the dough strength, with higher values indicating stronger dough. Stability time was significantly increased with the increasing of quinoa flour up to 20%. The increase in the stability time was related to the quantity of replacement. The dough sample containing 5-20% quinoa flour exhibited higher stability and resistance to mechanical mixing values than the control. The stability time in control sample (100% wheat flour) was 6.33±0.28 min, while blend containing 20% quinoa flour was 7.43±0.29 min; then stability time decreased in 25% and 30% quinoa flour to be 6.16±0.29 and 5.50±0.50 min, respectively. The results reported by [26] showed that blends containing 5 or 10% of quinoa flour showed good bread making properties, while blends with 15% of quinoa flour were not acceptable due to the lowering of dough stability time. In contrast to this investigation, the present study showed that the addition of quinoa flour up to 30% showed a good stability time against the control. In this case, we can emphasize that substitution of wheat flour with quinoa flour up to 30% had no negative effect on the rheological characteristics of dough. Value for subsequent stability of 5.5 min suggested that the dough might well perform under mechanical influences during the kneading and processing [6].

*The degree of softening* of wheat-quinoa flour blends was significantly increased as it was 91.67±2.88 and 145.67±5.00 BU in control and 30% quinoa flour, respectively. But there were no significant differences between control dough and dough containing quinoa flour up to 20%. The weakening or degree of softening in 25 and 30% quinoa flour was higher that other blends. This is probably due to the dilution of the protein network and the decrease of the gluten concentration which caused an increase in a degree of softening. These results are in agreement with earlier results of Abdelrahman [28] who found that addition of lupine flour to wheat flour at different amounts causes weakening in wheat dough and added that variation in hydration behaviour of two proteins may be reason for differences in dough characteristics.

#### 3.1.2. Extensograph Properties

Extensograph provides information about the viscoelastic behavior of dough. This equipment measures dough extensibility and resistance to extension. A combination of good resistance and good extensibility results is needed dough property [29]. Data in Table 3 showed rheological properties of dough with different ratios of quinoa flour by extensograph. The resistance of extension (elasticity) depends on the amount of gluten in dough. The elasticity was significantly decreased with the increasing of percentage of added quinoa flour. The wheat dough (control) (385.00±4.50 BU) and 5% QF dough (390.00±5.00) recorded the highest elasticity values among all other blends (from 10 to 30%), while 30% quinoa flour exhibited the lowest value (335.00±4.50 BU). There were no significant differences in the control (wheat dough) and 5% and 10% quinoa flour dough (145.00 ±3.00, 145.00 ±5.00, and 140.00 ±4.00 mm), respectively, which showed a higher extensibility values than other blends of quinoa flour, while the blends containing 25 and 30% quinoa flour exhibited the lowest extensibility values (105.00±3.00 and 108.00±4.14 mm), respectively. Elasticity was decreased with increasing of quinoa flour due to lake of gluten in quinoa flour (as gluten free). The variances in rheological properties among wheat flour and quinoa flour might commonly be attributed to the difference in chemical composition and structure of the constituents including protein and starch and their relations [30]. These findings are in agreement with earlier study of Enriquez et al. [26] who reported that addition of quinoa flour at ratio 5, 10, and 15% to wheat flour decreased the extensibility significantly. They also found a decrease of the extensibility due to the absence of gluten-like protein in quinoa flour. Generally, in the present study, quinoa flour showed slight effect on dough properties such as elasticity and extensibility.* Ratio number* is the ratio of elasticity/extensibility, which presented in Table 3. Increasing quinoa flour to wheat flour increased ratio number. There is no significant difference remarked among control and 5% and 10% quinoa flour, which showed lower ratio number than other blends of quinoa flour (2.65±0.12, 2.69±0.13, and 2.68±0.08, respectively). On the other hand, blend containing 25% quinoa flour showed the highest ratio number (3.33±0.18). Generally, quinoa flour is known to have weakening effect on dough properties. Our challenge in the present study was the ability to use quinoa flour in bread making at high amount up to 30% to increase the nutritional quality of bread without dramatic effect on rheological properties of dough.

### 3.2. Proximate Analysis of Quinoa Flour-Based Flat Bread

It is known that the addition of quinoa flour to wheat flour increases the nutritional value of final product. The chemical composition of the produced novel flat bread is presented in Table 4. The protein content in quinoa-based bread was increased gradually with increasing the percentage of quinoa flour from 12.12±0.63% in control to 15.85±0.065% in 30% quinoa flour. Increasing of protein content in bread referred to the high protein content of quinoa (20.03%). Incorporation of 30% of quinoa flour increased protein content by around 3.5%. Fat, ash, and crude fibre contents of the quinoa bread blends were also higher than their content in control, Table 4. The total carbohydrate content in quinoa bread blends (5% to 30%) was lower than control due to the content of protein, ash, and lipids in quinoa bread being higher than wheat bread. Results regarding bread making with quinoa were close to the data previously reported by Stikic et al. [6] who found that adding quinoa to wheat flour to produce pan bread led to increase the nutritive value and produce bread with higher content in protein, ash, fibre, and lipids than control made from wheat flour.

### 3.3. Minerals Content of Quinoa Flour-Based Flat Bread

Minerals content of flat bread made with different ratios of quinoa flour were present in Table 5. Significant differences of minerals content in control and all blends of quinoa flour were determined. Minerals content in 30% quinoa flour blend such as sodium (1582.13), potassium (88.47), magnesium (5.20), calcium (310.17), iron (23.18), copper (0.78), manganese (40.08), and zinc (4.33) were higher than other blends and control (100% wheat flour). Phosphorus was the only element to decrease gradually when quinoa flour increased; phosphorus in control was 103.97±0.27 mg/100gm while in 30% quinoa flour was 88.47±0.37 mg/100g. The increasing in those minerals in quinoa blends might be due to a high minerals content of quinoa than wheat. In the same time, [4, 31] showed that the minerals content of quinoa is about twice or three times than other cereals. These results also are in agreement with those reported by Ibrahium [32], who found higher content of minerals (Fe, Ca, and Zn) in biscuit contained quinoa flour than that made of wheat flour. Our findings might be useful in treatment of mineral deficiencies (especially iron, calcium, and zinc) that have a negative effect on human health and might lead to iron deficiency anaemia, rickets, osteoporosis, and diseases of the immune system [33, 34]. World Health Organization (WHO) estimated that anaemia affects over 2.5 billion people worldwide and indicated that iron deficiency anaemia is a significant problem throughout the world ranging from 1% in the industrialized countries to 56% in developing countries [35]. So, our quinoa-based bread could effectively contribute in the treatment of anaemia and malnutrition in our Egyptian community.

### 3.4. Amino Acids' Composition in Quinoa Flour-Based Flat Bread

The main characteristic of quinoa flour is the special quality of its amino acid composition. Hence, quinoa is one of the few plant foods that provide all essential amino acids for human life with values close to those set by FAO and its amino acid composition is similar to that of milk protein [36].  Table 6 illustrates the amino acids' composition of flat bread made with different percentages of quinoa flour (5-30%). Essential amino acids of threonine (0.46), valine (0.65), tyrosine (0.51), isoleucine (0.50), leucine (0.91), phenylalanine (0.65), histidine (0.36), lysine (0.49), and methionine (0.25) in 30% quinoa flour blend were higher than those in wheat flour bread (control). We noticed an increasing in those amino acids by adding quinoa flour to bread but cysteine was lower in quinoa blends compared to control; this was 0.30 g/100g in 30% quinoa flour but in control was 0.34. From the obtained data, flat bread made from quinoa flour had higher contents of all essential amino acids especially lysine than that made from wheat flour (control) with the exception of cysteine and proline. The increasing in those amino acids in quinoa blends might be due to the high quality of quinoa protein than wheat protein. Quinoa protein had essential amino acid contents equal to or beyond FAO/WHO/UNU [37] reference patterns, Table 6. Flat bread loaves made from quinoa flour were mainly high in lysine, which is the limiting amino acid in common cereals. This result is in agreement with previous results by Stikic et al. [6] who reported that lysine content in quinoa seeds is more than twofold higher than in wheat and also quinoa contains lysine 1.4 times more than soybean, 2.5-5.0 times more than corn, and 14.0 times more than milk. When compared to the requirements in school children and adults, quinoa protein can supply more than 150% the requirements of school children and more than 200% of adults [31]. Baking quality of flat bread loaves supplemented with quinoa flour is presented in Table 7. Significant differences (p≤0.05) were found in loaves weight among control and other all blends. The addition of quinoa flour resulted in a slight decrease in the loaf weight and loaf weight was 84.52±1.19 g for control, while the loaf weight was 81.13±0.88g for 30% QF, this variance in loaf weight might be due to extended baking time. There were significant differences in loaves volume between control and all other quinoa flour blends; on the contrary, there are no significant differences among other blends from 5 to 30% quinoa flour, Table 7. Loaves made of wheat flour showed the highest volume (191.33±7.63 cm^3^), while loaves made of 5% and 30% of quinoa flour showed the least volume (180.00 ±5.00 and 177.33±8.27cm^3^, respectively). These results are in contrast to the previous ones reported by [38, 39]. They reported that substitution of wheat flour by quinoa flour increased the loaf volume of bread compared to control. There are significant differences in specific volume of loaves among control and blends from quinoa flour. The specific volume of the control loaves was higher than that of loaves containing quinoa flour, where control loaf is 2.27±0.02 cm^3^ /g, while the loaves contain 30% quinoa flour which had the lowest loaf specific volume (2.14±0.03 cm^3^/g) but there is no significant difference observed between samples containing 5-30% of quinoa flour blends. This effect is might be due to the decreasing of viscoelasticity, which resulted from replacement of wheat flour with quinoa flour. These results were in agreement with Tomoskozi et al. [27] who reported that there was a significant decrease of the specific volume of breads prepared from wheat flour with higher (20–30%) contents of quinoa flour being observed. This is connected with the weakening of the gluten network in dough and reduced gas retention of dough. On the other hand, there were no significant differences noted in baking loss after baking for all blended samples. On the other hand, significant differences were found in roundness due to the manual baking practice but no significant differences in height of loaves were observed, Table 7.

### 3.5. Texture Properties of Quinoa-Based Flat Bread

The results of texture properties of wheat bread with different blends of quinoa flour (5-30%) are presented in Table 8. Loaves produced from quinoa flour blends were harder than control, and the hardness gradually increased with increasing of substitution levels. These results are in agreement with Park et al. [40] who reported that substitution of 30% quinoa flour led to poor extensible gluten network caused by hardening of bread crumb. Also, Iglesias-Puig et al. [41] reported that crumb hardness in pan bread increased when the ratio of quinoa in wheat-quinoa flour mixture increased from 25% to 50%. The current study was also close to the data obtained by Rosell [42] who found that textural hardness (firmness) in 100% quinoa flour bread was significantly (p ≤0.05) harder than the control. Loaves containing quinoa flour did not show any significant variation in resilience and springiness, while cohesiveness in wheat flour and 5% quinoa flour was slightly higher than in other blends (10-30 quinoa flour) due to the presence of prolamins contained gliadin in wheat flour 40–50% of the protein being extremely sticky and inelastic which resulted in the cohesiveness of dough. In the same respect, gumminess and chewiness increased with increasing of quinoa flour in blends. Increasing the gumminess and chewiness led to a slight deterioration of the organoleptic score in quinoa blends than wheat bread (control). These results are in agreement with results reported by Turkut et al. [13], who found that the chewiness increased in bread when quinoa flour amount increased against bread made of rice, potato, and buckwheat flour (as control).

### 3.6. Colour Properties

Colour characteristics quinoa-based flat bread: the colour parameters of bread obtained from quinoa flour blends are shown in Tables 9 and 10. Crust lightness (L) significantly decreased when the quinoa flours increased in the blends. The (L) value in control sample was 73.38±0.56, while in 30% quinoa flour it was 68.53±0.68. Loaves with higher concentration of quinoa flours had darker crust due to the dark colour of the quinoa flour and also increasing content of reducing sugars and proteins with lysine residues that react during baking producing non-enzymatic Maillard browning. The (a) value (redness) of crust loaves bread increased when quinoa flour increased due to nature of quinoa flour. The (a) value in control sample registered 2.87±0.14 while in 30% quinoa flour was 4.16±0.52. The (b) value (yellowness) for bread crust was also significantly increased when quinoa flour increased. The (b) value in control sample was 22.70±0.94, while in 30% quinoa flour was 25.57±1.12. These obtained results were close to the previous results by Stikic et al., [6] who stated that the bread containing quinoa flour (10, 15 and 20%) had yellow-reddish crispy crust. Similarly, Wang et al. [30] reported that increasing the amount of quinoa flour in the composite flour increased darkness and redness of bread. The increasing of darkness and redness of quinoa-based bread might be due to the high content of protein in quinoa flour, which resulted in the Maillard browning during baking. Colour of crumb of loaves made from different blends of quinoa flour was presented in Table 10; the (L) value showed significant reduction when increasing proportion of quinoa flours, yielding darker crumbs in quinoa flour-based flat bread. The (L) value in control sample was 66.5±0.03, while in 30% quinoa flour it was 60.75±0.08. The (a) value or redness was significantly increased when the proportion of quinoa flours was higher in the blends than wheat bread. The (a) value in control sample was 1.38±0.02 while in 30% quinoa flour it was 5.74±0.12. The (b) value also significantly increased when the ratio of quinoa flours was increased in the blends than wheat bread. The (b) value in control sample was 19.67±0.19, while in 30% quinoa flour it was 27.68±0.13. These results are in agreement with the earlier results by Lorenz and Coulter [39], who reported that lightness (L) value is lower in quinoa, while redness (a) and yellowness (b) values are higher in quinoa than wheat bread for crumb.

### 3.7. Sensory Evaluation of Quinoa Flour-Based Flat Bread

**Figure 1:**
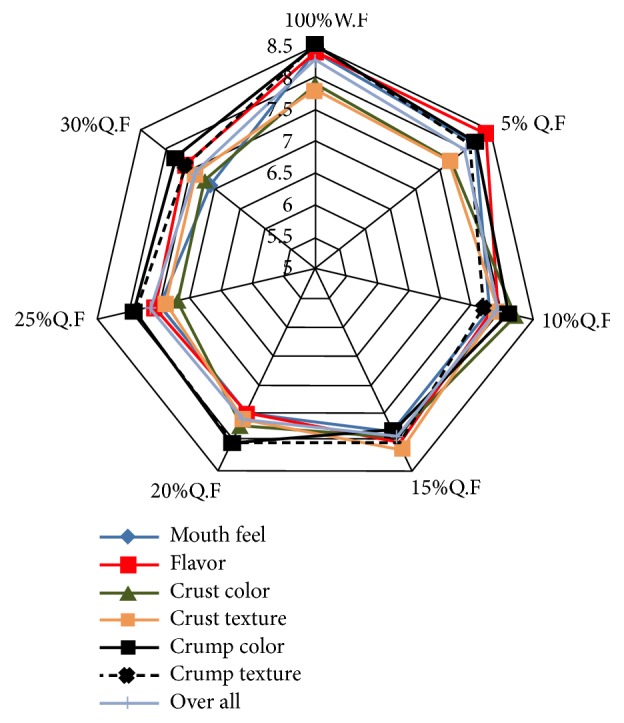
*Sensory evaluation of quinoa flour-based flat bread. ∗∗*WF = wheat flour; *∗∗∗*QF = quinoa flour. (1) Dislike extremely; (2) dislike very much; (3) dislike moderately; (4) dislike slightly; (5) neither like nor dislike; (6) like slightly; (7) like moderately; (8) like very much and like extremely (9).

Sensory evaluation of quinoa flour-based flat bread is the aspects of food experienced by the senses, including taste, sight, smell, and touch. The mean of sensory score of bread made from different ratio of quinoa flour was presented in Figure 1. It was clear that by increasing quinoa flour in the blend up to 30% the loaf shape had slightly changed but it was more acceptable. For mouth feel, there are significant differences among control and all blends, in which sensory score gradually decreased, the score in control was “like very much” while in 30% QF it was “like moderately”. The low score at high level of quinoa flour in bread may be due to the higher value of gumminess and chewiness in quinoa bread than wheat bread. The data also showed that flavour had higher score in case of control and 5, 10, and 15% of quinoa flour in blends, which resulted in good acceptance (like very much). For 20, 25, and 30% of quinoa flour in blends had score of 7.90 to 7.70, which is also accepted organoleptically (like moderately). When adding quinoa flour to wheat flour for making flatbread, it is important to note that very amazing flavour of quinoa (taste and odour) was perceived in all loaves of flat bread accompanied by quinoa flour. Taste of the loaves was unique, exceptional, nutty, and no bitterness aftertaste and very acceptable up to the 30% supplementation level. These results are in contrast with data obtained by Stikic et al. [6] who reported that pan bread containing quinoa up to 20% was slightly bitter, although it was acceptable. The disappearance of bitterness of produced quinoa-based bread in the present study might be due to our methodology in removing saponin from quinoa seeds. Overall acceptance in control and all blends with quinoa flour was significantly deferent. The overall acceptance in control was 8.24±0.49 while in 30% QF it was 7.48±0.57. This slight reduction in overall acceptance is due to the low score of mouth fell and dark colour of bread made from different ratio of quinoa flour. In the same time, the flat bread produced from wheat with different percentage of quinoa flour had a high organoleptic score (from like moderately to like very much). These results are in agreement with the results by Gordillo-Bastidas et al., who reported that replacement of refined flour by quinoa could produce changes in the sensory parameters of products, like darker colour due to the presence of bran, and may influence the consumer decisions. In the same time, Linnemann and Dijkstra [9] reported the sensory evaluation in baking products of flavour, texture, and appearance to be moderately acceptable, a crunchy texture, a unique shape, and a nutty or wheaty flavour.

## 4. Conclusion

**Figure 2:**
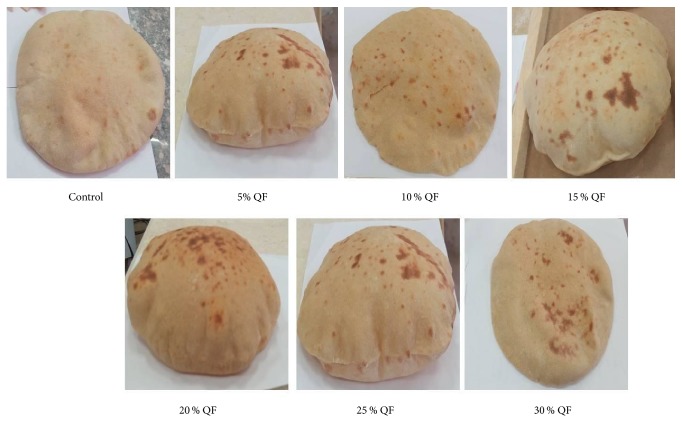
Quinoa flour-based flat bread.

The present study showed that the flat bread supplemented with different levels of quinoa flour (5%, 10%, 15%, 20%, 25%, and 30%) increased its levels of proteins, essential amino acids, crude fiber, and minerals that was nutritionally superior to wheat bread (as control), Figure 2. Flat bread supplemented with quinoa flour had higher contents of all essential amino acids especially lysine which is very low in wheat flour. Minerals content in 30% quinoa flour blend (sodium, potassium, magnesium, calcium, iron, copper, manganese, and zinc) were higher than control (100% wheat flour), which is very important for treatment of malnutrition. The addition of quinoa flour did not dramatically affect the rheological characteristics of dough. The substitution of wheat flour with quinoa flour had a slight effect on rheological properties but did not cause deforming of dough. So, these effects can be neglected compared to the raising of the nutritional value. Sensory characteristics of bread were excellent even at 30% supplementation level. Modification of technological procedure of seed preparation afforded the inclusion of such high levels of quinoa flour in bread making possible the development of novel highly nutritive baking products which might be contributed in the resolve of the anaemia and malnutrition problems in developing countries.

## Figures and Tables

**Table 1:** Recipe of flat bread supplemented with quinoa flour (g/100g).

Ingredients	Blends						
	C*∗*	5%	10%	15%	20%	25%	30%
		QF*∗∗*	QF	QF	QF	QF	QF
Wheat flour	100	95	90	85	80	75	70

Quinoa flour	0	5	10	15	20	25	30

Yeast (gm)	1.5	1.5	1.5	1.5	1.5	1.5	1.5

Salt (gm)	1	1	1	1	1	1	1

Water (ml)	59.5	60.5	61	62	62	63	64

*∗*C= 100% wheat flour; *∗∗*QF = quinoa flour.

**Table 2:** Farinograph parameters of dough supplemented with quinoa flour.

Bread formula	Water absorption %	Arrival time (min)	Dough development time (min)	Stability time (min)	Degree of softening (B.U)
Control	59.50^f^ ±0.50	1.50^a^ ±0.01	3.33^c^ ±0.29	6.33^b^ ±0.28	91.67^b^ ±2.88

5%	60.50^e^ ±0.50	1.50^a^ ±0.00	4.16^b^ ±0.29	6.50^b^ ±0.29	91.66^b^ ±2.87

10%	61.17^d^ ±0.29	1.50^a b^ ±0.29	4.83^a^ ±0.28	7.33^a^ ±0.29	98.33^b^ ±2.89

15%	62.16^c^ ±0.28	1.50^a b^ ±0.26	4.83^a^ ±0.28	7.36^a^ ±0.29	91.67^b^ ±2.89

20%	62.17^c^ ±0.29	1.45^ab^ ±0.28	4.66^ab^ ±0.29	7.43^a^ ±0.29	100.00^b^ ±5.00

25%	63.17^b^ ±0.29	1.45^ab^ ±0.21	4.66^ab^ ±0.29	6.16^b^ ±0.29	115.00^ab^ ±5.00

30%	63.83^a^ ±0.29	1.45^b^ ±0.30	4.16^b^ ±0.30	5.50^c^ ±0.50	145.67^a^ ±5.00

Values with different letter in the same column were significantly different at *p* ≤ 0.05.

**Table 3:** Extensograph parameters of supplemented with quinoa flour.

Bread formula	Elasticity (B.U)	Extensibility (mm)	R.N *∗*	Energy (Cm^2)^
Control	385.00^a^ ±4.50	145.00^a^ ±3.00	2.65^c^ ±0.12	88.33^a^ ±1.53

5%	390.00^a^ ±5.00	145.00^a^ ±5.00	2.69^c^ ±0.13	83.33^a^ ±2.89

10%	375.00^b^ ±5.50	140.00^a^ ±4.00	2.68^c^ ±0.08	70.00^b^ ±5.00

15%	370.00^b^ ±4.00	125.00^b^ ±5.00	2.96^b^ ±0.07	56.67^c^ ±2.88

20%	355.67^c^ ±5.64	115.00^c^ ±3.00	3.09^b^ ±0.15	46.66^d^ ±2.88

25%	350.00^c^ ±5.00	105.00^d^ ± 3.00	3.33^a^ ±0.18	46.68^d^ ±2.89

30%	335.00^d^ ±4.50	108.00^cd^ ±4.14	3.05^b^ ±0.16	43.33^d^ ±2.88

Values with different letters in the same column indicate significant differences (*P*≤ 0.05). *∗*Ratio number = (Elasticity/ Extensibility).

**Table 4:** Chemical composition of quinoa flour-based flat bread (on dry weight basis).

Bread formula	%Ash	%Protein	%Lipids	%Crude Fiber	%Carbohydrates	Calories (kcal)
Control	1.58^f^ ±0.09	12.12^f^ ±0.63	1.19^g^ ±0.03	0.54^f^ ±0.07	84.56^a^ ±0.18	397.45^a^ ±0.81

5%	2.11^e^ ±0.08	13.71^e^ ±0.06	1.39^f^ ±0.02	0.66^f^ ±0.05	82.11^b^ ±0.11	395.89^b^ ±0.55

10%	2.31^d^ ±0.04	14.36^d^ ±0.21	1.68^e^ ±0.02	0.85^e^ ±0.06	80.80^c^ ±0.12	395.75^b^ ±0.20

15%	2.68^c^ ±0.03	14.78^c^ ±0.02	1.87^d^ ±0.03	1.05^d^ ±0.04	79.61^d^ ±0.03	394.37^bc^±0.13

20%	2.70^bc^ ±0.04	15.14^b^ ±0.20	2.02^c^ ±0.04	1.31^c^ ±0.11	78.83^e^ ±0.30	394.12^c^ ±0.75

25%	2.74^b^ ± 0.06	15.64^a^ ±0.07	2.42^b^ ±0.05	1.57^b^ ±0.07	77.63^f^ ±0.13	394.83^b^ ±0.35

30%	2.82^a^ ±0.05	15.85^a^ ±0.06	2.85^a^ ±0.06	1.86^a^ ±0.03	76.61^g^ ±0.20	395.52^b^ ±0.53

Values with different letters in the same column indicate significant differences (*P*≤ 0.05).

**Table 5:** Minerals content of quinoa flour-based flat bread (mg /100 g).

Minerals mg/100gm	Control	5%	10%	15%	20%	25%	30%
Na	1460.42^g^ ±0.73	1484.01^f^ ±0.32	1486.67^e^±0.43	1507.72^d^ ±0.33	1550.91^c^ ±0.14	1566.24^b^±0.70	1582.13^a^ ±1.30

K	355.90^e^ ±1.15	379.16^d^±0.42	380.54^d^±0.46	390.86^c^±0.60	392.53^b^ ±0.52	393.21^b^ ±0.22	406.36^a^ ±0.60

Mg	36.64^d^ ±0.22	36.63^d^ ±0.22	36.59^d^ ±0.12	37.44^c^ ±0.21	38.49^b^ ±0.19	40.02^a^ ±0.14	40.08^a^ ±0.18

Ca	197.53^g^±0.09	265.62^f^ ±0.12	273.16^e^ ±1.48	280.37^d^ ±1.83	298.85^c^ ±0.09	304.42^b^ ±0.07	310.17^a^ ±.12

P	103.97^a^ ±0.27	102.25^b^±0.11	100.30^c^±0.26	100.29^c^±0.41	95.73^d^ ±0.40	92.01^e^ ±0.30	88.47^f^ ±0.37

Fe	16.14^e^ ±0.01	20.55^d^±0.08	20.51^d^±0.07	21.45^c^ ±0.11	21.96^b^±0.10	22.88^a^ ±0.03	23.18^a^ ±0.03

Cu	0.51^e^ ±0.01	0.61^d^ ±0.01	0.59^d^ ±0.01	0.66^c^ ±0.02	0.70^b^±0.03	0.72^b^ ±0.05	0.78^a^ ±0.05

Mn	3.17^e^ ±0.02	3.60^c^±0.03	3.62^c^±0.01	3.63^c^±0.03	4.47^b^ ±0.02	4.95^a^ ±0.02	5.20^a^ ±0.03

Zn	2.61^e^ ±0.02	2.98^d^ ±0.01	3.11^d^ ±0.01	3.81^c^ ±0.01	3.79^c^ ±0.03	3.85^b^ ±0.04	4.33^a^ ±0.05

Values with different letters in the same row indicate significant differences (*P*≤ 0.05).

**Table 6:** Amino acids content of quinoa flour-based flat bread (g /100 g).

Amino Acids (%)	Control	5%	10%	15%	20%	25%	30%
E. A. As *∗*							

Threonine	0.35	0.39	0.34	0.36	0.36	0.41	0.46

Valine	0.57	0.64	0.46	0.48	0.46	0.63	0.65

Tyrosine	0.46	0.47	0.43	0.42	0.43	0.50	0.51

Isoleucine	0.44	0.45	0.40	0.40	0.40	0.48	0.50

Leucine	0.81	0.84	0.76	0.75	0.74	0.88	0.91

Phenylalanine	0.59	0.61	0.57	0.56	0.54	0.65	0.65

Histidine	0.29	0.30	0.27	0.29	0.29	0.34	0.36

Lysine	0.29	0.34	0.24	0.28	0.28	0.42	0.49

Cysteine	0.34	0.29	0.25	0.25	0.20	0.37	0.30

Methionine	0.18	0.22	0.19	0.20	0.19	0.24	0.25

NEA.As*∗∗*							

Aspartic	0.64	0.67	0.57	0.63	0.63	0.87	0.87

Serine	0.46	0.60	0.51	0.52	0.51	0.53	0.66

Glutamic	3.72	3.92	3.50	3.31	3.12	3.71	3.72

Glycine	0.51	0.52	0.46	0.47	0.47	0.62	0.62

Alanine	0.49	0.52	0.40	0.41	0.42	0.59	0.62

Arginine	0.52	0.62	0.61	0.67	0.71	0.82	0.91

Proline	1.41	1.44	1.31	1.23	1.20	1.31	1.30

*∗*EAAs: essential amino acids; *∗∗* NEAAs: non-essential amino acids, where blends: (control)=100% wheat flour.

**Table 7:** Baking quality characteristics of quinoa flour-based flat bread.

Bread formula	Weight (g)	Volume (Cm^3^)	Specific volume (Cm^3^/g)	Baking loss %	Roundness (cm)	Height (cm)	Baking time (Sec.)
Control	84.52^a^ ±1.19	191.33^a^ ±7.63	2.27^a^ ±0.02	20.48^ab^ ±1.88	17.63^b c^ ±0.55	7.83^a^±0.76	115

5%	82.20^b c^ ±1.37	180.00^b^ ±5.00	2.19^ab^ ±0.01	22.03^a^ ±2.66	19.47^a^ ±0.45	8.00^a^ ±0.50	115

10%	81.43^c^ ±1.29	179.00^b^ ±5.00	2.20^ab^ ±0.01	22.10^a^ ±2.26	19.00^ab^ ±0.00	8.00^a^ ±1.00	120

15%	81.28^b c^ ±1.62	178.01^b^ ±5.00	2.19^ab^ ±0.02	22.56^a^ ±1.35	17.67^b c^ ±0.58	9.00^a^ ±1.00	125

20%	81.16^b c^ ±1.61	176.33^b^ ±7.64	2.17^ab^ ±0.01	18.09^b^ ±0.55	17.97^abc^ ±0.95	8.67^a^ ±0.58	125

25%	82.07^b^ ±1.05	176.00^b^ ±5.00	2.14^b^ ±0.02	21.57^a^ ±0.98	17.97^abc^ ±1.05	8.63^a^ ±0.55	130

30%	81.13^c^ ±0.88	177.33^b^ ±8.27	2.18^ab^ ±0.03	21.52^a^ ±1.34	17.00^c^ ±1.73	7.83^a^ ±0.29	130

Values with different letters in the same column indicate significant differences (*P*≤ 0.05). WF = wheat flour; QF = quinoa flour.

**Table 8:** Texture properties of quinoa flour-based flat bread.

Texture Parameters	Control	5%	10%	15%	20	25%	30%
Hardness (g)	137.67^e^±13.65	139.33^e^ ±14.01	161.33^c^ ±3.21	164.67^cd^±13.80	181.00^bc^ ±11.27	191.00^b^±3.61	216.00^a^ ±6.56

Cohesiveness	0.96^a^ ±0.05	0.91^ab^ ±0.04	0.86^bc^ ±0.06	0.82^c^ ±0.01	0.83^c^±0.01	0.82^c^ ±0.02	0.83^c^ ±0.05

Springiness (mm)	2.76±0.05^a^	2.75^a^ ±0.05	2.68^a^ ±0.02	2.66^a^ ±0.06	2.57^ab^ ±0.33	2.51^ab^ ±0.08	2.36±0.21^b^

Resilience	0.37^a^±0.09	0.34^a^±0.05	0.35^a^±0.04	0.33^a^±0.01	0.36^a^±0.03	0.28^a^±0.03	0.28^a^±0.05

Gumminess (g)	131.92^c^±17.76	127.65^c^±18.85	139.38^bc^±11.93	134.37^bc^±9.49	150.17^bc^±7.95	156.61^b^±4.11	179.95^a^±10.71

Chewiness (mJ)	3.40±0.10^e^	3.63±0.14^de^	3.93^d^ ±0.06	4.43^c^ ±0.31	5.36^b^ ±0.34	6.02^a^ ±0.12	6.24^a^ ±0.33

Values with different letters in the same row indicate significant differences (*P*≤ 0.05).

**Table 9:** Color parameters of crust of quinoa flour-based flat bread.

Bread formula	Color parameter		
	L	a	b
Control	73.38^a^ ±0.56	2.87^b^ ±0.14	22.70^b^ ±0.94

5%	72.32^a^ ±0.35	3.08^b^ ±0.02	23.93^a^ ±0.45

10%	70.37^b^ ±0.53	4.67^a^ ±0.55	24.85^a^ ±1.21

15%	68.77^bc^ ±2.16	4.38^a^ ±0.78	25.15^a^ ±2.57

20%	67.90^c^ ±0.35	4.14^a^ ±0.14	24.60^a^ ±0.84

25%	67.40^c^ ±0.68	4.32^a^ ±0.10	25.08^a^ ±0.92

30%	68.53^c^ ±0.68	4.16^a^ ±0.52	25.57^a^ ±1.12

Values with different letter in the same column were significantly different at p≤0.05. L is lightness; a is redness; b is yellowness.

**Table 10:** Color parameters of crumb of quinoa flour-based flat bread.

Bread formula	Color parameter		
	L	a	b
Control	66.5^a^ ±0.03	1.38^g^ ±0.02	19.67^f^ ±0.19

5%	66.15^b^ ±0.06	1.75^f^ ±0.08	22.43^e^ ±0.08

10%	66.53^a^ ±0.09	2.32^e^ ±0.04	23.72^d^ ±0.14

15%	64.28^c^ ±0.22	3.12^d^ ±0.08	24.58^c^ ±0.36

20%	62.83^d^ ±0.05	3.27^c^ ±0.07	25.63^b^ ±0.14

25%	62.15^e^ ±0.17	4.73^b^ ±0.07	27.75^a^ ±0.10

30%	60.75^f^ ±0.08	5.74^a^ ±0.12	27.68^a^ ±0.13

Values with the different letter in the same column were significantly different at *p*≤0.05. L is lightness; a is redness; b is yellowness.

## Data Availability

The data used to support the findings of this study are available from the corresponding author upon request.
